# Supplementary material for: Landscape of somatic single nucleotide variants and indels in colorectal cancer and impact on survival
Source: Nat Commun. 2020 Jul 20;11:3644. doi: 10.1038/s41467-020-17386-z (PMC7371703; doi:10.1038/s41467-020-17386-z)
Supplement: Supplementary file 1 — Supplementary Information [file 41467_2020_17386_MOESM1_ESM.pdf]

SUPPLEMENTARY INFORMATION:

Landscape of somatic single nucleotide variants and indels in colorectal cancer and impact on survival

Zaidi et al.

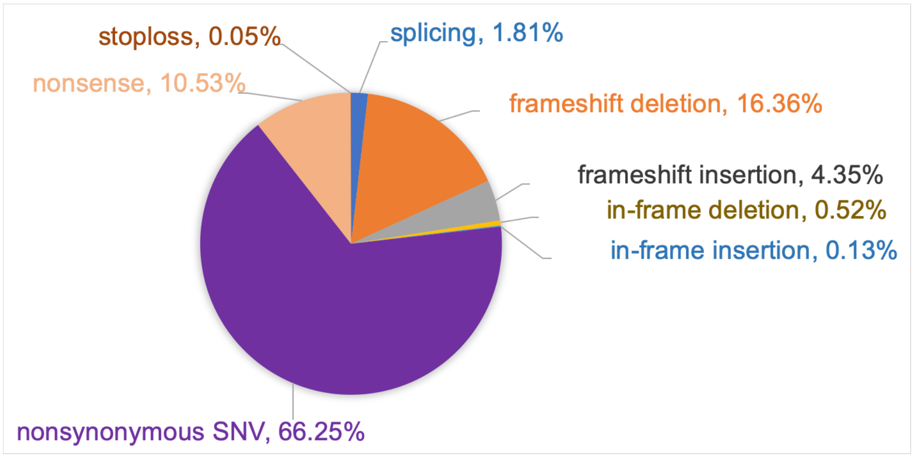

Supplementary Figure 1. Classification of non-silent mutations found in 2,105 tumors. Most frequent mutations are nonsynonymous SNVs (Single Nucleotide Variants).

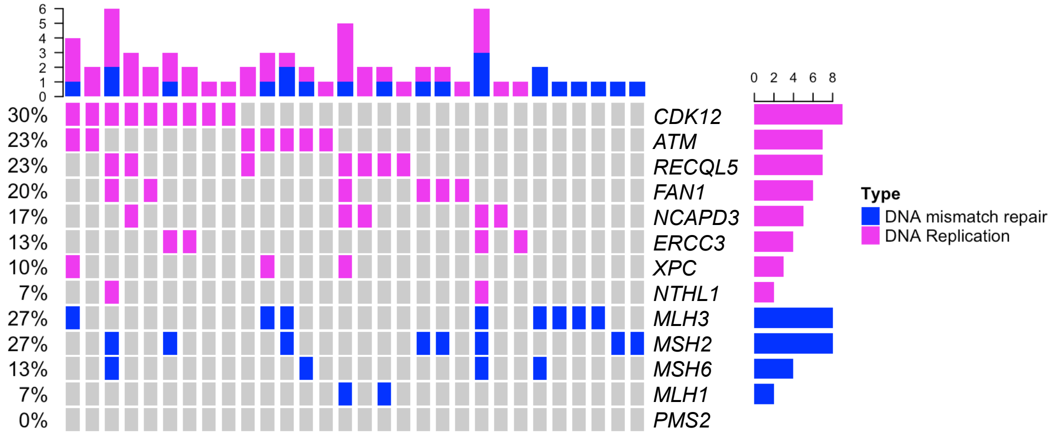

Supplementary Figure 2. Mutations in genes involved in DNA replication or repair in hypermutated MSS tumors without mutations in the *POLE* and *POLD1* genes. Mutations in DNA replication or DNA mismatch repair are shown in different colors.

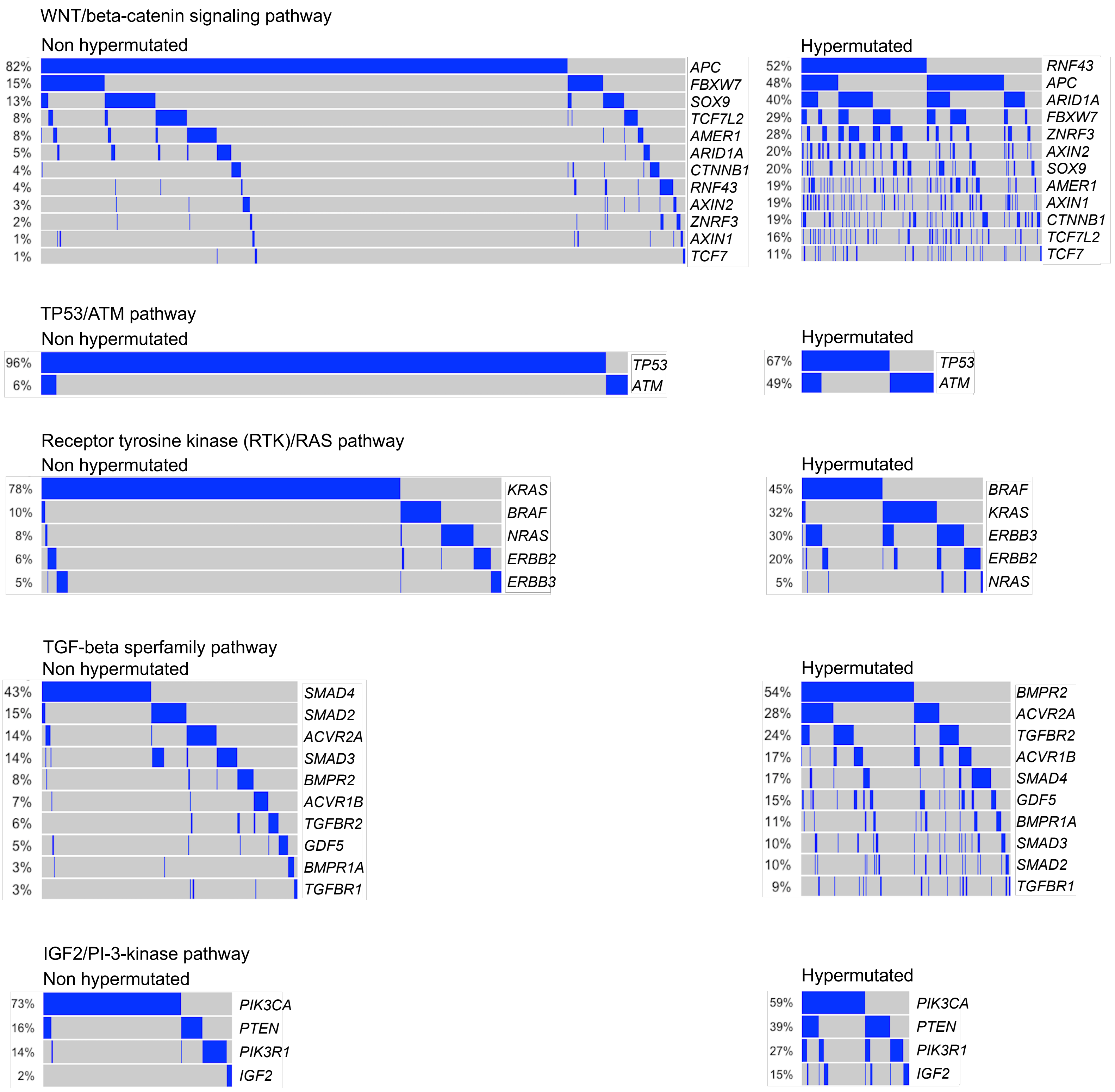

Supplementary Figure 3. Contributions of genes with somatic mutations in selected pathways in non-hypermutated and hypermutated tumors. Only cases with mutations are shown.

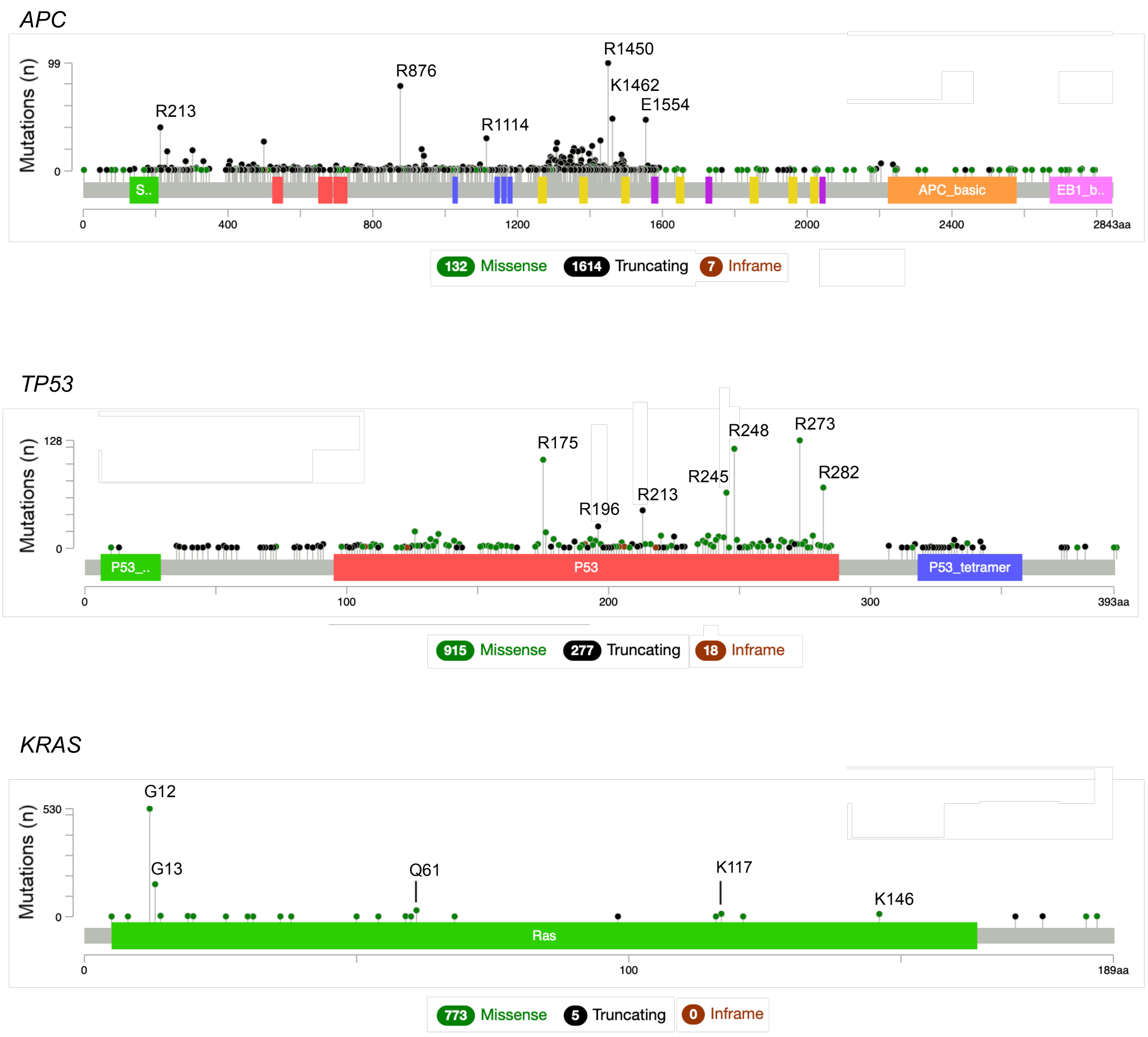

Supplementary Figure 4. Distribution of somatic mutations in *APC*, *TP53*, and *KRAS* mutated tumors. Green, black, and brown circles denote missense mutations, truncations, and in-frame indels, respectively. Colored boxes show various domains of the proteins. The Mutation Mapper tool from cBioPortal was used for plots.

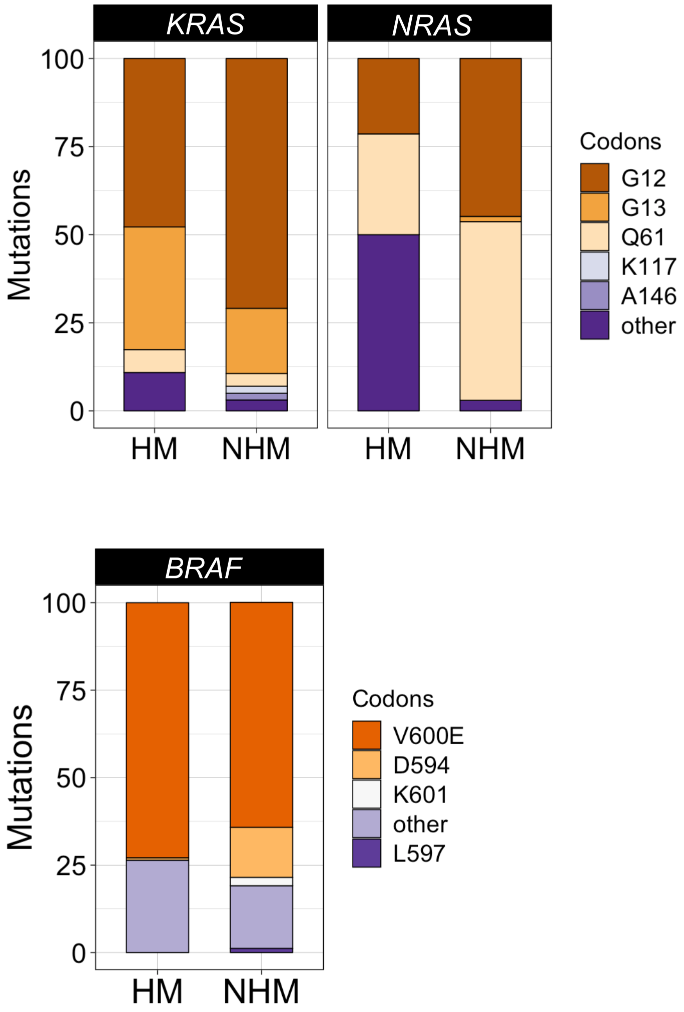

Supplementary Figure 5. Codons affected by recurrent mutations in *KRAS*, *NRAS*, and *BRAF*. Hypermutated (HM) and non-hypermutated (NHM) tumors exhibit differences in mutated codons in these genes.

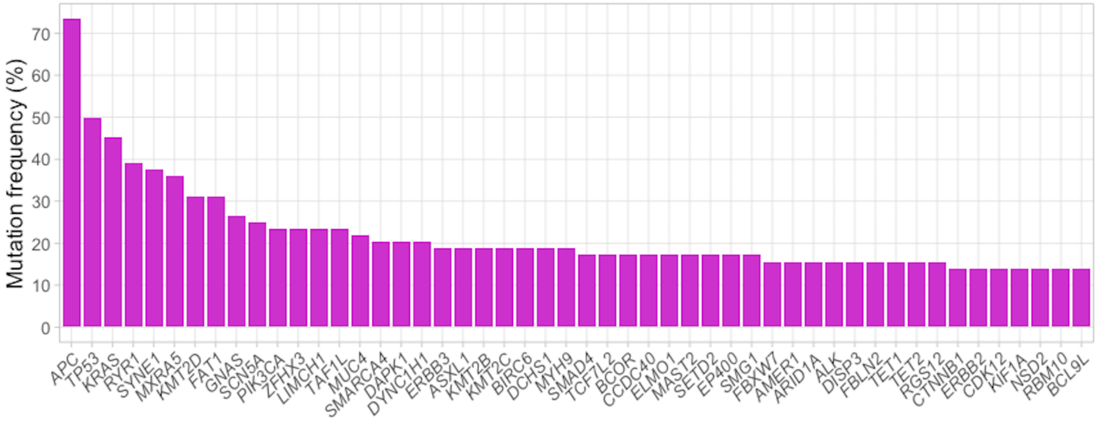

Supplementary Figure 6. Non-silent mutations in 64 MSS-hypermutated tumors without mutations in the *POLE* and *POLD1* genes. The top 50 mutated genes are shown.

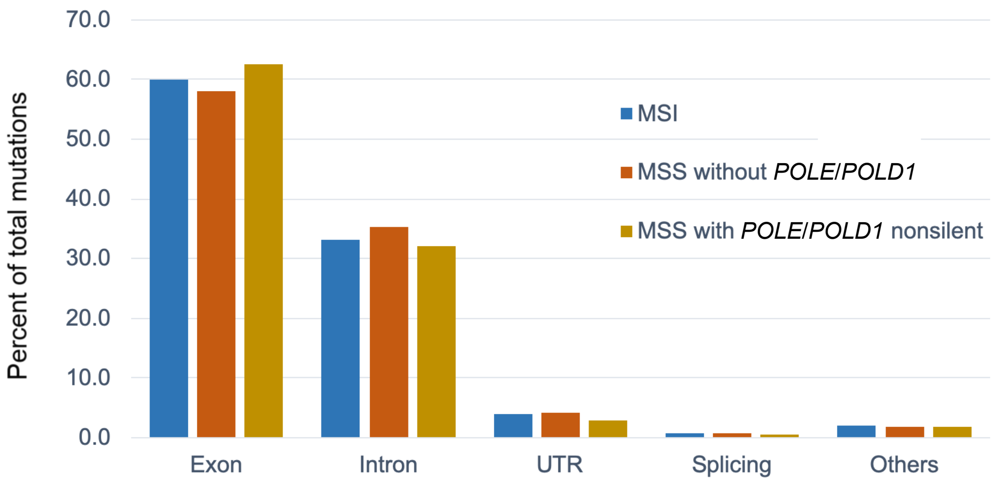

Supplementary Figure 7. Mutations types across hypermutated tumors, with MSI, or MSS tumors with and without nonsilent mutations in *POLE/POLD1*. UTR = untranslated regions of transcripts; Others = intergenic, upstream, downstream, and non-coding regions in the targeted sequencing panel.

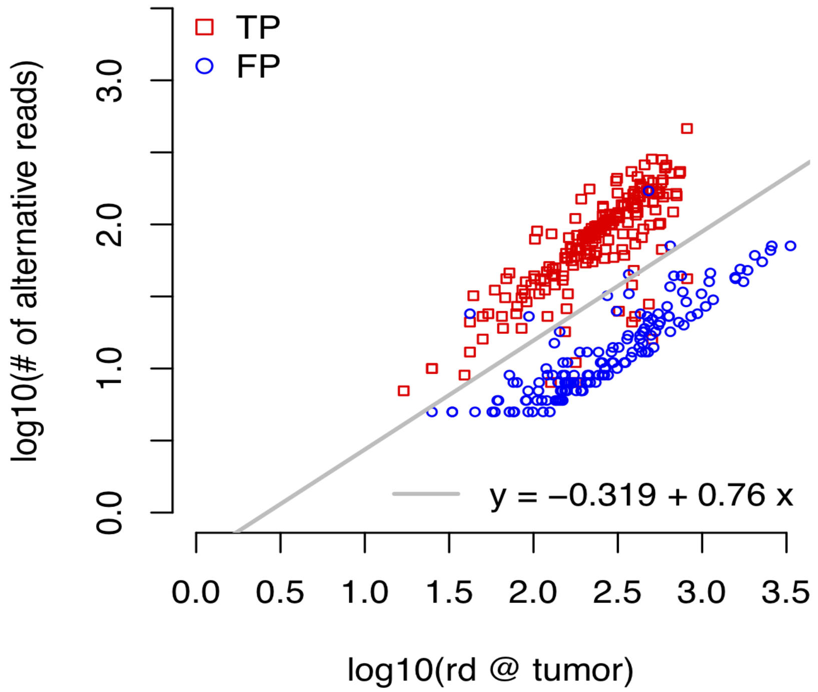

Supplementary Figure 8. Separation of False Positive (FP) and True Positive (TP) somatic SNV calls. Data was plotted using log10 (read-depth in tumor) and log10 (alt read depth) for somatic SNVs from all 7 dilution samples.

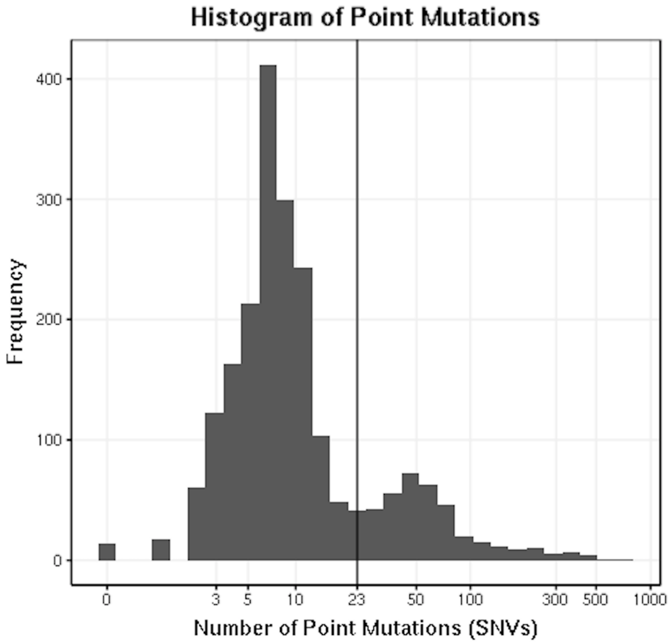

Supplementary Figure 9. Histogram of point mutations (SNVs) called in 2,105 colorectal tumors in the log-scale. The threshold to define a hypermutated tumor was set to 23.

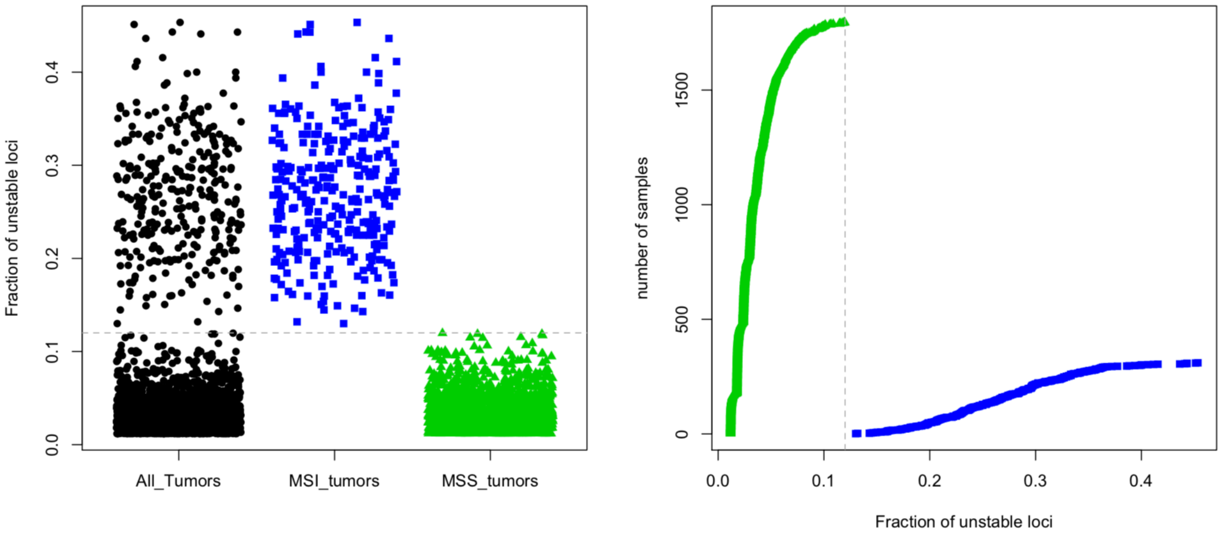

Supplementary Figure 10. Defining the microsatellite status of tumors. The fraction of unstable microsatellite loci identified by the targeted panel (left) and cumulative distribution of that fraction (right). We decided on 12% unstable sites threshold to define MSI status, indicated by the dashed line. Blue = MSI, green = MSS.

SUPPLEMENTARY METHODS

1. Design for the targeted sequencing panel

To ensure the most comprehensive selection of putative relevant genes and other somatic mutations related to colorectal cancer (CRC) we used multiple sources and approaches to select the targeted sequencing panel. We included 700 whole exome sequencing paired normal-tumor (formalin-fixed paraffin embedded, FFPE) samples from Health Professional Follow up Study (HPFS) and the Nurses' Health Study (NHS) and 525 whole exome sequencing paired normal-tumor (fresh frozen) samples from The Cancer Genome Atlas (TCGA) colon and rectal adenocarcinoma data [1–3]. These two datasets were separately called and analyzed for somatic mutations. Analyses were conducted stratified by hypermutated cases (defined as >=17 SNVs/Mb) vs non-hypermutated samples. A primary inclusion criterion for selecting a gene was that the gene was significantly mutated (P<0.01 based on MutSigCV, details below) in non-hypermutated cases in either the TCGA samples or the HPFS/NHS samples. Furthermore, we conducted pathway analysis based on the results from MutSigCV to identify CRC-related pathways and additional genes within relevant pathways. We conducted nonrandom clustering analysis to identify additional genes with the gain of function mutations not identified otherwise. We evaluated the list of significantly mutated genes for the hypermutated samples based on HPFS/NHS and TCGA; however, as the list was very large and likely included a large number of false positive findings we only included a gene if additional evidence was available, such as relative low p-value in non-hypermutated samples, support from the literature review or being located in highlighted pathways. In addition to these individual level data analyses, we conducted a literature search [4–13] and evaluated genes listed in COSMIC (http://cancer.sanger.ac.uk/cosmic) to identify additional genes. These approaches led to the selection of 205 genes. As MSI testing is possible with the next generation sequencing we included 236 MSI/homopolymer markers. As a quality control measure, we included a gender marker.

Prioritizing genes for inclusion in the targeted panel using MutSigCV

We ran MutSigCV on the Mutation Annotation Format (MAF) files separately for HPFS/NHS and TCGA samples (both stratified by hypermutated vs non-hypermutated) using the MutSigCV package as described in [14]. We used the covariates file provided by the Broad that accounts for the effect of DNA replication time, chromatin state (open/closed), and general level of transcription activity on the background mutation rate for each gene. To generate an accurate coverage file we used the exome kits, VCRome 2.1 and Roche v2, used on the samples run on Solid (subset of TCGA samples) and Illumina respectively, taking the union of the exons included in each kit to define the gene, and the reading frame for each exon provided by the UCSC Genome Browser for the corresponding RefSeq transcript. We used the HUGO gene names and the RefSeq transcripts provided in the MAF. The genes to add to the panel were selected from the list of significantly mutated genes based on MutSigCV with a P-value < 0.01.

Pathway analysis

The HPFS/NHS and TCGA data was used to include genes connected to the main pathways involved in CRC which did not make the above-prioritized list of genes. A total of 19 additional genes were added from the datasets of non-hypermutated and hypermutated tumors with P-values of <0.1 and <0.01, respectively.

Using non-random clustering to identify genes with gain of function mutations to add to targeted panel

For the TCGA samples, we applied the nonrandom clustering method as described in [15] using the longest RefSeq isoform stratified by hypermutated vs non-hypermutated samples. This resulted in a list of significant clusters of point mutations. If a gene contained a cluster with a p-value < 0.01, then this gene was included in the panel. Genes with significant clusters corresponding to single amino acids were given particular consideration for the panel.

We also did nonrandom clustering restricted to indels to identify clusters of indels for consideration for the panel. Since many of the clusters corresponded to indels at homopolymers in the hypermutated set of samples, likely resulting from MSI, we considered this set separately and included genes containing indels at homopolymer that occurred in at least 5 samples.

2. Somatic single nucleotide variants (SNVs) calling and filtering

SNV calling

Samples passing QC were selected for somatic mutation calling. We called somatic SNVs for each sample using both Strelka (version 1.0.15) and MuTect (version 1.1.7).

We modified the default configuration of Strelka by removing the read-depth filter isSkipDepthFilters=1, according to the recommendation for analyzing exome/targeted sequencing data using Strelka (https://sites.google.com/site/strelkasomaticvariantcaller/home/faq).

Both Strelka and Mutect require the reference genome sequence. In addition, Mutect also uses input of mutation annotation from COSMIC and dbSNP. We obtained these files from the following locations.

- hg19 reference from UCSC Genome Browser website
  http://hgdownload.cse.ucsc.edu/goldenPath/hg19/bigZips/.
- Cosmic somatic mutations
  We downloaded Cosmic somatic mutation vcf files for coding and non-coding variants (CosmicCodingMuts.vcf.gz and CosmicNonCodingVariants.vcf.gz) from Cosmic website (sftp-cancer.sanger.ac.uk/cosmic/grch37/cosmic/v76/VCF). Then sort these files according to the reference genome and merge them to one vcf file.
- dbSNP annotations
  The dbSNP annotations (version 138) were downloaded from GATK bundle (ftp://gsapubftp-anonymous@ftp.broadinstitute.org/bundle/), and sorted according to the reference genome.

We annotated the somatic mutation calls by ANNOVAR to obtain the functional consequence of somatic mutations, e.g., intergenic, intronic, or exonic, and if exonic, whether it is synonymous, missense, start gain/loss etc. In addition, ANNOVAR also provides annotation of alternative allele frequency in Exome Aggregation Consortium

(ExAC) version 0.3 while excluding TCGA studies (ExAC_nontcga_ALL). Such ExAC alternative allele frequency was used later for filtering potential germline mutations.

SNV filtering

Initial filtering

We retained a somatic SNV if it has a PASS status from both Strelka and MuTect or PASS from Strelka, and fail from MuTect only because of "clustered_read_position". We rescued mutations that failed the "clustered_read_position" filter because the sequencing reads are clustered due to the design of our amplicon sequencing strategy. Customized filters on clustered reads were applied in the following steps:

- Poor mapping quality, defined as more than 2% of covering reads have a mapping quality of 0
- Strand bias, defined as a MuTect LOD score ratio of the forward and reverse strand to be >1000 or < 1e-3
- MAF in ExAC to be > 1e-4
- Read-depth in tumor or normal < 25 reads or < 5 reads supporting the alternative allele
- Clustered read position: candidate SNV occurred in the first or second base of either end of reads supporting the mutation

In addition to original sequencing run with default (100%) reagent concentration, we conducted three additional sequencing runs with 100%, 75%, and 50% dilution of reagent for 7 samples. We found the dilution of reagent does not have a significant impact on the number of somatic mutation calls and decided to use 50% diluted reagents in the following studies. Ignoring the factor of dilution, we essentially obtained 4 replicates for 7 samples.

We first use these replicates to evaluate the set of initial filtering criterion. As shown in Supplementary Table 2, most SNVs that are removed by our filters are called in only one of the four replicates, except the ExAC filter. It is expected that the mutations filtered by ExAC filter appear in multiple replicates because they are likely germline mutations.

Further filtering based on read-depth and variant allele frequency

We consider the mutations that appear once in the four replicates as False Positives (FPs), and the mutations that appear in all four replicates as True Positives (TPs). We seek to classify FPs and TPs. To remove redundancy of TPs (since one mutation is called in four replicates), we chose the one in 50% dilution to represent the four replicates of each TP. Our conclusion of FP/TP classification is robust to this choice. After the aforementioned initial round of filtering of SNVs, there are 147 SNVs appearing in one replicate, and 204 appear in four replicates.

We manually examined the distributions of different characteristics of the remaining SNVs, such as read depth in tumor or normal samples, mutation call confidence score reported by MuTect or Strelka, and the number of alternative reads (i.e., the reads harboring alternative allele). It turns out that the FPs and TPs can be well separated in the two-dimensional space of log10(read depth in tumor) and log10(number of alternative reads) (Supplementary Figure 8). We applied linear discriminant analysis (LDA) (R function MASS/lda) to derive the classification rule: somatic SNV call is TP if and only if

$\log_{10}$(alt read depth) > -0.319 + 0.76*$\log_{10}$(tumor read-depth).

Our samples can be divided into two groups based on read-depth in the paired normals. In the first batch, read-depth in paired normals is comparable to read-depth in tumors (median 522 and IQR [254, 851]), while the second batch, the read-depth in paired normal is significantly reduced (median 60 and IQR [35, 100]). To study the somatic mutation calling and filtering, we down-sampled the read-depth in paired normals of dilution samples to 1/10 of the original depth.

Next, we applied the same steps of somatic mutation calling and initial filtering and then applied LDA to classify TP and FP somatic mutations. The resulting classification rule is

$\log_{10}$(alt read depth) > -0.225 + 0.73*$\log_{10}$(tumor read-depth).

We applied one of these two LDA filtering depending on the read-depth in paired-normal samples.

<u>Amplicon artifact filtering</u>
Sanger sequencing of a subset of candidate mutations revealed another source of false-positives: amplicon-specific mismatches. These false positives may occur during clonal amplification on the flow cell or during addition of fluorescently tagged nucleotides, which results in miscalled bases on either ends of the reads, but it is not limited to it. We identified strong amplicon artifacts present in any position of the reads, likely introduced during PCR-based library preparation. Hence, trimming candidates located in the first n bases of either end of the reads is considered both wasteful and insufficient. Instead, we decided to compare the number of reads supporting the alternative allele and number of reads supporting the reference allele between overlapping amplicon clusters -i.e., reads derived from the same primer pair covering a candidate mutation. This was determined by every read pair's alignment start position.

In other words, for each mutation, we generate a contingency C x 2 table, where C is the number of clusters and the two columns of this table correspond to the number of reference and alternative reads. Resulting contingency tables were evaluated by two methods and a candidate gets removed if both methods vote to reject:

χ^2 test of a contingency table if the total number of amplicon clusters is >1 and p-value is smaller than 5e-5 vote to reject.

Cumulative sum, which is an ad-hoc statistic resembling Kolmogorov–Smirnov test statistic to compare two distributions. Here we compare the read count distributions between reference and alternative alleles. We apply the following steps to a contingency table to calculate this statistic:

1. Order rows in a contingency table by the number of alternative read counts from high to low.
2. Calculate cumulative sum of alternative and reference read counts.
3. Divide each row by the total number of alternative and reference reads respectively (i.e., divide by the last row of the cumulative sum).
4. Subtract reference read fraction from alternative read fraction for each cluster.
5. Calculate a statistic d as the maximum of the alternative read fraction subtracted by the reference read fraction.

We removed a mutation if d > 0.35 and the number of clusters was >1.

All “remove candidates” with more than two clusters were then tested again, but the cluster with the lowest VAF was removed to avoid over filtering due to misaligned reads.

3. Short somatic InDel calling and filtering

InDel calling

To call somatic InDels, we used a majority-win approach, which requires two out of three callers, VarScan2 (2.4.3), VarDict (Feb 2017) and Strelka (1.0.15) to agree on an initial set of candidate InDels.

VarScan2 input was generated from SAMtools (1.8) mpileup. It was configured to keep anomalous read pairs and per-Base Alignment Quality (BAQ) computation was disabled. Anomalous read pairs have parts of the forward and reverse read overlapping. BAQs are not expected by VarScan2 and, hence, not needed. Additionally, mapping qualities were adjusted by 50, as recommended by SAMtools and reads with a mapping quality of 3 or less were excluded. VarScan2 was configured to call somatic InDels with a minimum variant allele frequency of 0.05, a somatic p-value of < 0.05, strand-filter enabled (InDels with >90% strand bias are removed), minimum read coverage of 25 in tumor and normal and an expected tumor purity of 70%. Subsequently, VarScan2’s list of InDels was filtered for somatic status (SS) to be 2.

VarDict was set up in paired variant calling mode with a minimum variant allele frequency of 0.05 and minimum mapping quality of 3. The minimum number of variant supporting reads was set to 4 and the highest variant allele frequency in normal was set to 1%. Subsequently, variant status (STATUS) was filtered for germline or any mutation type that was not a deletion or an insertion.

Strelka was configured to skip the depth filter, as recommended for target sequencing data. Additionally, variants with allelic frequency of < 0.05 were removed from Strelka’s pass-filter output list.

If two of the three callers called the same InDel (same locus and alternative allele), the variant was considered as candidate for further filtering or was removed otherwise.

Candidate list was annotated by ANNOVAR to obtain further functional information, such as impact prediction and population frequencies. Annotated candidates that were called off-target were also removed prior filtering.

InDel filtering

Candidates were filtered for false-positives to obtain a final list of somatic InDels by applying the filter steps listed below. To keep coverage-based filtering consistent, per-base coverage profiles for every position covered by the target amplicon panel, was generated using bam-readcount (https://github.com/genome/bam-readcount). Subsequently for each candidate, the somatic InDel VAF was obtained from # of alt-supporting reads divided by # of alt-supporting reads + # of reference-supporting reads.

Coverage and VAF filters

A candidate is removed if the minimum total depth is less than 25x in normal and less than 200x in tumor. The number of reads supporting the alternative allele needs to be 10 or higher. We also require a VAF ≥ 0.1 in the tumor. For indels with 0.1 ≤ VAF < 0.2, we require a minimum total depth of 500x in tumor.

Background filters

We noticed background signals of alternative reads for indel calls in normal samples. More specifically, when examining the number of alternative reads versus the total number of reads in normal samples, the number of alternative reads increased linearly with increasing total read-depth. Such background, when appearing in a tumor, may lead to false positive indel calls. Therefore, we applied the following additional filters.

Before background filtering, there are 15,021 indel calls from 4,920 unique indels. A unique indel is defined based on its location, reference allele, and alternative allele, and a unique indel may occur in multiple samples.

For each unique indel, we obtained the alternative read count and total read-depth in all normal or tumor samples (regardless of being called in that sample or not). Then summarized such read counts using different percentiles as well the mean value of Variant Allele Frequency (VAF) as estimated by fitting a beta-binomial distribution on alternative read counts given total read counts.

Using these statistics, we divided all 4,920 unique indels into three groups and every alternative allele at each position was tested separately.
The first group includes 2,145 (~44%) unique indels that have alternative alleles in only one or two normals, with maximum VAF in normals <10%. This is the group of indels for which we did not apply additional filters.

The second group includes 222 unique indels that were potential germline indels. We removed them in all samples. More specifically, an indel is a potential germline indel if Maximum VAF in normals >= 0.25, or 99.9 percentile >= 0.2, or mean VAF in normals >= 0.02.

The remaining 2,553 unique indels had some background signals. We removed an indel call in a tumor sample if the alternative read count was not significantly larger than expected from the background distribution. For each indel, we obtained a background beta-binomial distribution fitted using the data from the normal samples. Then, for each tumor sample, we assessed the tail probability that the observed read count in this tumor sample may arise from the background beta-binomial distribution. If the tail probability is $<10^{-5}$, we kept the indel, otherwise it was discarded.

After such background filtering, we obtained 11,986 indels that correspond to 4,432 unique indels.

4. Quality control metrics

To assess the quality of sequencing data, we examined various quality metrics using Picard (2.18.29). Median insert size -that is the number of base pairs between sequencing adapters of paired reads- for each tumor library was calculated using CollectInsertSizeMetrics. Hybrid-selection (HS) metrics for each tumor library were collected using CollectHsMetrics. HS metrics were used to calculate Read pass filter aligned ratio (PCT_PF_READS), AT drop out (AT_DROPOUT), GC drop out (GC_DROPOUT) and Percent target bases at 20X in tumor (PCT_TARGET_BASES_20X). Chimeric pairs ratio was determined from the tool CollectAlignmentSummaryMetrics (PCT_CHIMERAS). The OxoG Q-score was obtained from the PreAdapterSummaryMetrics of Picard's CollectSequencingArtifactMetrics tool. The OxoG Q-score is the scaled probability for any given G:T transversion in a sample to be an artifact rather than a true mutation. Samples with OxoG Q score <35 are expected to have adverse impact on variant calling[16]. The lowest value among those samples passing QC filters was 44. The C>T variant combination proportion was calculated using the number of C>T mutations divided by the total number of SNV mutations after QC filtering. Because mutational burden, MSI status and mutations in *POLE*/*POLD1* have substantial impact on the overall mutational profile, we show the quality control metrics stratified by these variables in Supplementary Table 3. The quality of the data is fairly consistent among groups defined by mutational burden, MSI status, and mutations in *POLE/POLD.*

5. Description of participating studies

Colorectal Cancer Study of Austria (CORSA)
In the ongoing CORSA study, more than 16,000 Caucasian participants have been recruited within the province-wide screening project "Burgenland Prevention Trial of Colorectal Disease with Immunological Testing" (B-PREDICT) since 2003 [17]. All inhabitants of the Austrian province Burgenland aged between 40 and 80 years are annually invited to participate in fecal immunochemical testing and haemoccult positive screening participants are invited for colonoscopy. CORSA participants have been recruited in the four KRAGES hospitals in Burgenland, Austria, and additionally, at the Medical University of Vienna (Department of Surgery), the Viennese hospitals "Rudolfstiftung" and the "Sozialmedizinisches Zentrum Süd", and at the Medical University of Graz (Department of Internal Medicine).

Cancer Prevention Study II (CPS-II)
The CPS-II Nutrition Survey cohort is a prospective study of cancer incidence and mortality in the United States, established in 1992 and described in detail elsewhere [18,19]. At enrollment, participants completed a mailed self-administered questionnaire including information on demographic, medical, diet, and lifestyle factors. Follow-up questionnaires to update exposure information and to ascertain newly diagnosed cancers were sent biennially starting in 1997. Reported cancers were verified through medical records, state cancer registry linkage, or death certificates. The Emory University Institutional Review Board approves all aspects of the CPS II Nutrition Cohort.

Darmkrebs: Chancen der Verhütung durch Screening (DACHS)
This German study was initiated as a large population-based case-control study in 2003 in the Rhine-Neckar-Odenwald region (southwest region of Germany) to assess the

potential of endoscopic screening for reduction of colorectal cancer (CRC) risk and to investigate etiologic determinants of disease, particularly lifestyle/environmental factors and genetic factors [20,21]. Cases with a first diagnosis of invasive CRC (International Classification of Diseases 10 codes C18-C20) who were at least 30 years of age (no upper age limit), German speaking, a resident in the study region, and mentally and physically able to participate in a one-hour interview, were recruited by their treating physicians either in the hospital a few days after surgery, or by mail after discharge from the hospital. Cases were confirmed based on histologic reports and hospital discharge letters following diagnosis of CRC. All hospitals treating CRC patients in the study region participated. Based on estimates from population-based cancer registries, more than 50% of all potentially eligible patients with incident CRC in the study region were included. During an in-person interview, data were collected on demographics, medical history, family history of CRC, and various life-style factors, as were blood and mouthwash samples. Formalin-fixed, paraffin-embedded, surgical specimens of CRC patients were collected from cooperating pathology institutes and transferred to the tissue bank of the National Center for Tumor Diseases in Heidelberg.

Colon Cancer Registry (CCFR) [22].

The CCFR is an NCI-supported consortium consisting of six centers dedicated to the establishment of a comprehensive collaborative infrastructure for interdisciplinary studies in the genetic epidemiology of colorectal cancer. The CCFR includes data from approximately 42,500 total subjects in 15,000 families (10,500 probands, and 26,770 unaffected and affected relatives and 4,276 unrelated controls and 923 spouse controls). Cases and controls, age 20 to 74 years, were recruited at the six participating centers beginning in 1998. Between 1999 and 2002, female cases and controls 50-74 years enrolled into the Seattle CCFR (SCCFR) were subsequently enrolled in a complementary study of post-menopausal hormone use and CRC risk (PMH). The CCFR and PMH implemented a standardized questionnaire that was administered to all participants, and included established and suspected risk factors for colorectal cancer, including questions on medical history and medication use, reproductive history (for female participants), family history, physical activity, demographics, alcohol and women who also enrolled tobacco use, and dietary factors. This study selected tumor samples for these molecular subtypes study from two of the CCFR sites, Ontario and Seattle (including a subset of the PMH study).

Supplementary Table 1. Study population characteristics.

Characteristic	Sequencing data (n)	Mean age at diagnosis*	Follow up data (n)	CRC specific deaths (n)	Median survival time** (CI)
Study population					
CORSA	171	67	0	0	NA
CPS-II	537	75	535	79	93.8 (85.4-102.7)
DACHS	200	69	198	31	60.6 (60.2-60.7)
OFCCR	674	58	514	89	194.1 (192.5-196)
SCCFR	523	58	433	117	170.9 (166.1-175.5)
Sex					
Female	1038	64	838	148	143.7 (133-155.3)
Male	1067	64	842	168	132.6 (123.2-146.5)
Tumor cancer site					
Right-sided	900	66	773	138	126.4 (113.2-139.8)
Left-sided	1184	62	900	177	148.2 (136.7-159.8)
Tumor stage					
Stage 1 or local	545	67	486	19	128.1 (116.4-138.9)
Stage 2 or 3 or regional	1228	64	1041	201	145.5 (132.6-156.7)
Stage 4 distant	188	59	115	86	135.3 (89.8-190.1)
Tumor hypermutation status					
Non-hypermutated	1710	63	1340	290	148.4 (139.8-159.6)
Hypermutated	395	67	340	26	95.7 (83.1-112.5)
Tumor MSI status					
MSS	1795	63	1397	300	147 (138.7-157.3)
MSI	310	67	283	16	104.1 (90-117.2)

* age at diagnosis reported in years.
** Median survival time (in months) based on reverse KM estimator [23].

Supplementary Table 2. The number of somatic SNVs removed by each filter, grouped by the number of times a mutation is called.

Filter name	Number of times a mutation is called			
	1	2	3	4
Poor mapping	16	1	0	0
Strand Bias	7	0	0	0
Clustered Position	146	14	2	0
ExAC MAF*	5	0	1	20
Alternative reads	16	1	0	1
Tumor Read depth	4	0	0	0

* Minor Allele Frequency in Exome Aggregation Consortium (ExAC) database.

Supplementary Table 3. Quality control metrics stratified by groups defined by hypermutation status, microsatellite status, and *POLE*/*POLD1* mutation status.

Quality control metrics	Group 1*	Group 2*	Group 3*	Group 4*	All
Number of samples	1,710	63	268	64	2,105
Age of samples (mean)**	14.68	14.11	14.14	13.73	14.57
Insert size (median)	123.1	120.8	122.7	120.7	122.9
Read pass filter aligned ratio	0.996	0.989	0.996	0.993	0.996
Chimeric pairs ratio	0.031	0.042	0.036	0.049	0.032
AT drop out (%)	7.63	13.57	7.27	13.20	7.93
GC drop out (%)	3.95	2.42	4.31	2.02	3.89
Percent target bases at 20X in tumor	94.65	89.7	94.64	90.09	94.36
C>T variant combination (%)	0.56	0.59	0.61	0.56	0.56
OxoG Q-score (mean)	79.41	75.46	80.63	72.14	79.45

*Group 1: NHM, Group 2: HM-MSS-*POLE* or *POLD1* mutated, Group 3: HM-MSI, Group 4: HM-MSS-non-*POLE* or *POLD1* mutated; HM = hypermutated, NHM = non-hypermutated, MSS = microsatellite stable, MSI = microsatellite instability

** Age defined as year of sequencing minus year of diagnosis

SUPPLEMENTARY REFERENCES

1. Giannakis, M. *et al.* Genomic Correlates of Immune-Cell Infiltrates in Colorectal Carcinoma. *Cell Rep.* **15,** 857–865 (2016).
2. Grasso, C. S. *et al.* Genetic mechanisms of immune evasion in colorectal cancer. *Cancer Discov.* **8,** 730–749 (2018).
3. Cancer Genome Atlas Network. Comprehensive molecular characterization of human colon and rectal cancer. *Nature* **487,** 330–337 (2012).
4. Bellido, F. *et al.* POLE and POLD1 mutations in 529 kindred with familial colorectal cancer and/or polyposis: review of reported cases and recommendations for genetic testing and surveillance. *Genet. Med.* **18,** 325–332 (2016).
5. Kandoth, C. *et al.* Mutational landscape and significance across 12 major cancer types. *Nature* **502,** 333–339 (2013).
6. Guda, K. *et al.* Inactivating germ-line and somatic mutations in polypeptide N-acetylgalactosaminyltransferase 12 in human colon cancers. *Proc Natl Acad Sci USA* **106,** 12921–12925 (2009).
7. Ngeow, J. *et al.* Prevalence of germline PTEN, BMPR1A, SMAD4, STK11, and ENG mutations in patients with moderate-load colorectal polyps. *Gastroenterology* **144,** 1402–9, 1409.e1 (2013).
8. Lawrence, M. S. *et al.* Discovery and saturation analysis of cancer genes across 21 tumour types. *Nature* **505,** 495–501 (2014).
9. Yang, H. *et al.* Meta-analysis of the rs4779584 polymorphism and colorectal cancer risk. *PLoS ONE* **9,** e89736 (2014).
10. Tuupanen, S. *et al.* Identification of 33 candidate oncogenes by screening for base-specific mutations. *Br. J. Cancer* **111,** 1657–1662 (2014).
11. Nahorski, M. S. *et al.* Investigation of the Birt-Hogg-Dube tumour suppressor gene

(FLCN) in familial and sporadic colorectal cancer. *J. Med. Genet.* **47,** 385–390 (2010).

12. Fabregat, A. *et al.* The Reactome Pathway Knowledgebase. *Nucleic Acids Res.* **46,** D649–D655 (2018).
13. Rohlin, A. *et al.* GREM1 and POLE variants in hereditary colorectal cancer syndromes. *Genes Chromosomes Cancer* **55,** 95–106 (2016).
14. Lawrence, M. S. *et al.* Mutational heterogeneity in cancer and the search for new cancer-associated genes. *Nature* **499,** 214–218 (2013).
15. Ye, J., Pavlicek, A., Lunney, E. A., Rejto, P. A. & Teng, C.-H. Statistical method on nonrandom clustering with application to somatic mutations in cancer. *BMC Bioinformatics* **11,** 11 (2010).
16. Stewart, C., Leshchiner, I., Hess, J. & Getz, G. Comment on “DNA damage is a pervasive cause of sequencing errors, directly confounding variant identification”. *Science* **361,** (2018).
17. Hofer, P. *et al.* MNS16A tandem repeats minisatellite of human telomerase gene: a risk factor for colorectal cancer. *Carcinogenesis* **32,** 866–871 (2011).
18. Calle, E. E. *et al.* The American Cancer Society Cancer Prevention Study II Nutrition Cohort: rationale, study design, and baseline characteristics. *Cancer* **94,** 2490–2501 (2002).
19. Campbell, P. T. *et al.* Establishment of the cancer prevention study II nutrition cohort colorectal tissue repository. *Cancer Epidemiol. Biomarkers Prev.* **23,** 2694–2702 (2014).
20. Brenner, H. *et al.* Reduced risk of colorectal cancer up to 10 years after screening, surveillance, or diagnostic colonoscopy. *Gastroenterology* **146,** 709–717 (2014).
21. Jia, M. *et al.* No association of CpG island methylator phenotype and colorectal cancer survival: population-based study. *Br. J. Cancer* **115,** 1359–1366 (2016).

22. Newcomb, P. A. *et al.* Colon Cancer Family Registry: an international resource for studies of the genetic epidemiology of colon cancer. *Cancer Epidemiol. Biomarkers Prev.* **16,** 2331–2343 (2007).

23. Schemper, M. & Smith T. A note on quantifying follow-up in studies of failure time. *Control Clin. Trials.* **17**(4):343-346 (1996).
