# Supplementary material for: Landscape of somatic single nucleotide variants and indels in colorectal cancer and impact on survival
Source: Nat Commun. 2020 Jul 20;11:3644. doi: 10.1038/s41467-020-17386-z (PMC7371703; doi:10.1038/s41467-020-17386-z)
Supplement: Supplementary file 3 — Description of Additional Supplementary Files [file 41467_2020_17386_MOESM3_ESM.pdf]

**Description of Additional Supplementary Files**

File Name: Supplementary Data 1
Description: variant calls

File Name: Supplementary Data 2
Description: Survival analyses among patients with gene-level or pathwaylevel somatic mutations by hypermutation status.

File Name: Supplementary Data 3
Description: Distribution of somatic mutated genes and pathways by tumor site, adjusted for MSI status and mutation burden.

File Name: Supplementary Data 4
Description: Distribution of somatic mutated genes and pathways by tumor stage, adjusted for MSI status and mutation burden.

File Name: Supplementary Data 5
Description: Distribution of somatic mutated genes and pathways by sex, adjusted for MSI status and mutation burden.

File Name: Supplementary Data 6
Description: Clinical Attributes.
